# Editorial: Bioconversion of insect resources for sustainability

**DOI:** 10.3389/fbioe.2024.1448572

**Published:** 2024-07-15

**Authors:** Jun Wang, Carlos L. Cespedes-Acuña, Zhaojun Wei

**Affiliations:** ^1^ Jiangsu Key Laboratory of Sericultural and Animal Biotechnology, School of Biotechnology, Jiangsu University of Science and Technology, Key Laboratory of Silkworm and Mulberry Genetic Improvement, Ministry of Agricultural and Rural Affairs, The Sericultural Research Center, Chinese Academy of Agricultural Sciences, Zhenjiang, Jiangsu, China; ^2^ Department of Basic Sciences, Faculty of Sciences, University of Bío-Bío, Chillan, Chile; ^3^ School of Food and Biological Engineering, Hefei University of Technology, Hefei, China

**Keywords:** insect resources, bioconversion, sustainability and health, novel food process, food security and nutrition

In recent years, the concept of bioconversion of insect resources has gained traction as a promising solution to address the pressing issues of food security, environmental sustainability, and resource scarcity. Often overlooked as a potential food source, insects offer a viable and eco-friendly alternative that could revolutionize food production and consumption. The bioconversion of insect resources involves the utilization of insects as a source of protein and other nutrients. Insects are incredibly efficient at converting organic matter into high-quality protein, making them a highly sustainable and cost-effective food source (van et al., 2023). One of the key benefits of bioconversion of insect resources is its potential to alleviate the strain on our planet’s resources. With the global population projected to reach 9.7 billion by 2050, the demand for food is expected to increase significantly (Lähteenmäki-Uutela et al., 2021; Rivero-Pino et al., 2024). By incorporating insects into our diets, we can reduce the pressure on traditional livestock production systems and mitigate the environmental impact of agriculture. The European Food Safety Authority (EFSA) has given the go-ahead for several insects to be sold as food in Europe. These insects include the migratory locust (*Locusta migratoria*, 48.5% of protein), the house cricket (*Acheta domesticus*, 60.3% of protein), and the yellow mealworm (*Tenebrio molitor*, 57% of protein) (Scaffardi and Formici, 2022). Bioconversion of insect resources presents a unique opportunity to promote biodiversity and conservation efforts. Despite the numerous benefits of bioconversion of insect resources, there are still challenges that need to be addressed. One of the main hurdles is overcoming the cultural stigma associated with consuming insects in many parts of the world (Omuse et al., 2024). Innovations in insect farming technology, such as automated systems and sustainable feed sources, are essential to maximize the potential of insect resources for food production. Collaboration between researchers, policymakers, and industry stakeholders is key to advancing the bioconversion of insect resources and integrating them into mainstream food systems (Lin et al., 2023). In summary, the topic aims to explore the potential of using insects as a sustainable resource for various applications, including utilizing insects for their protein content, converting organic waste into valuable products through insect bioconversion, and promoting the use of insects as an eco-friendly alternative to traditional livestock farming.

As a result of the sericulture sector, the current efficiency in utilizing resources of silkworm pupae is relatively low. Bioactive peptides are generated from proteins through enzymatic hydrolysis and this approach not only addresses the issue of resource utilization but also enhances the production of nutritionally valuable additives. Ge et al. investigated the effect of tri-frequency ultrasonic pretreatment (22/28/40 kHz) on enzymatic hydrolysis of silkworm pupae protein and reported that ultrasonic pretreatment significantly increased the hydrolysis kinetic efficiency as well as the surface hydrophobicity, thermal stability, crystallinity and antioxidant activities of the hydrolysate. Thus, the utilization of tri-frequency ultrasonic pretreatment may serve as a promising strategy for augmenting enzymatic hydrolysis and enhancing the functional characteristics of silkworm pupae protein.

Numerous studies have been carried out regarding the application of transgenic silkworms and their innate spinning mechanism to produce spider silk fibers with superior properties. Nonetheless, investigations focusing on the utilization of non-spider biological proteins to enhance the mechanical characteristics of silk fibers through the molecular configuration of silk proteins remain limited. Dai et al. researched transgenic silkworms expressing the *Drosophila* dumpy to produce silk with high mechanical properties by constructing donor plasmids containing fusion genes of *Fib-H Dumpy* and *FibL-Dumpy*. Notably, an increase in *β*-sheet secondary structure was observed among increases in maximum stress, toughness, and elastic recovery rate. The findings demonstrated the effective incorporation of two external gene expression cassettes, controlled by internal promoters, into the genome of the silkworm through the utilization of piggyBac-mediated transgenic techniques. Therefore, *Drosophila* Dumpy significantly enhanced the silk properties, presenting a valuable opportunity for leveraging various biological proteins in the development of superior silk fibers.

According to Su et al., the intestinal microbiota of longhorn beetles could serve as promising candidates for pest management strategies, particularly in the context of utilizing lignocellulosic materials through either disruption or utilization of their cellulose-degrading capabilities. After screening the bacterial diversity of cellulose-degrading bacteria in the gut of *Glenea cantor* (Fabricus) larvae via molecular and bioinformatic techniques targeting the V3 and V4 regions, a total of 563,201 valid sequences and 814 OTUs were obtained. The analysis of microbial diversity indicated a significant presence of diverse bacteria, with emphasis on the essential role of gut bacteria in the host’s physiological processes. Specifically, the study identified nine genera of intestinal bacteria with cellulose degradation capabilities, with five belonging to the genus *Pseudomonas*, underscoring their critical function in the breakdown of cellulose. Thus, these findings provide important information on potential strategies for pest management using internal digestion and cellulose-degrading bacteria in longhorn beetles as targets.

As a result of several variabilities and uncertainties in characterizing feed efficiencies of the black soldier fly (BSF), *Hermetia illucens* which is employed in entomoremediation processes due to the high efficiency of its larvae, metabolic performance models were used to predict the growth efficiency of BSF larvae. Eriksen N. summarized the metabolic performance and feed efficiency of BSF. The review aimed to demonstrate how measuring the metabolic performance of BSF larvae can enhance our comprehension of the larvae’s contribution to entomoremediation processes. The carbon equivalent and mass balance as performance indicators revealed greater variability considering more feed substrates other than chicken feed. Also, it was reported that the efficiency of feed assimilation and growth rates, as well as the costs associated with growth, maintenance, and larval lifespan, have been demonstrated to impact the conversion of feed into growth by BSF larvae.

According to Kobelski et al., black soldier fly (BSF), *Hermetia illucens* serves as a valuable protein source for animal feed, however, the mechanism of reproduction and the amount of eggs produced are issues that restrict the industrial rearing of BSF. They developed a model-based process to optimize the production of BSF eggs via controlled environmental variables to stimulate and enhance the adult BSF life cycle. Through literature data fitting and simulations, the calculated trajectories for environmental variables demonstrated higher output and shorter production cycles at reasonable energy costs compared to the standard approach. This model-based optimization when applied to different scenarios, showcases the practicality and adaptability of the developed model to enhance the rearing practices of BSF through environmental stimulation, leading to potential improvements in egg production efficiency. The utilization of this technology is exemplified across a range of scenarios which exhibits pragmatic applicability of the constructed model. Thus, through model-based process optimization, researchers can fine-tune the production process and identify potential bottlenecks or areas for improvement. This approach can help to streamline operations, reduce costs, and increase the overall efficiency of BSF egg production, making it a valuable tool for sustainability.
